# Mixed T Cell Chimerism After Allogeneic Hematopoietic Stem Cell Transplantation for Severe Aplastic Anemia Using an Alemtuzumab-Containing Regimen Is Shaped by Persistence of Recipient CD8 T Cells

**DOI:** 10.1016/j.bbmt.2016.11.003

**Published:** 2017-02

**Authors:** Francesco Grimaldi, Victoria Potter, Pilar Perez-Abellan, John P. Veluchamy, Muhammad Atif, Rosemary Grain, Monica Sen, Steven Best, Nicholas Lea, Carmel Rice, Antonio Pagliuca, Ghulam J. Mufti, Judith C.W. Marsh, Linda D. Barber

**Affiliations:** 1 Department of Haematology, King's College Hospital NHS Foundation Trust, London, United Kingdom; 2 Department of Clinical Medicine, Haematology Division, AOU Federico II, Naples, Italy; 3 Division of Cancer Studies, King's College London, London, United Kingdom

**Keywords:** Hematopoietic stem cell transplantation, Alemtuzumab, Aplastic anemia, T cells, Chimerism

## Abstract

Prevention of graft-versus-host disease (GVHD) is paramount for allogeneic hematopoietic stem cell transplantation (HSCT) to treat nonmalignant diseases. We previously reported that allogeneic HSCT for severe aplastic anemia (SAA) using the fludarabine, cyclophosphamide, and alemtuzumab (Campath-1H) (FCC) regimen is associated with a very low risk of GVHD and excellent clinical outcomes. We now report a single-center study of 45 patients with longer follow-up and investigation of lymphocyte recovery. Overall survival (OS) was 93%, and event-free survival (EFS) was 90.7%. Acute and chronic GVHD each occurred in 6 patients (13.3%), and only 1 case was severe. Mixed T cell chimerism was frequent and persisted after cessation of immunosuppression. T cells were extensively depleted, representing only 11.3% of lymphocytes at day 30 and rising to 43.8% by 1 year, but still significantly below normal levels (67.2%; *P* = .018), and deficiency persisted after immunosuppressive therapy (IST) withdrawal. Depletion of CD4 T cells was particularly profound, causing inversion of the normal CD4:CD8 T cell ratio. T cell subset composition was also abnormal, with memory and effector T cells predominating for at least 6 months after FCC HSCT. Analysis of T cell subset chimerism showed that CD4 T cells were predominantly donor-derived at 1 year, whereas recipient-derived CD8 T cells shaped mixed chimerism with a notable contribution of recipient effector CD8 T cells. The prolonged mixed T cell chimerism after IST withdrawal and low incidence of GVHD indicates the establishment of mutual tolerance, but the low incidence of viral disease suggests maintenance of antiviral immunity. Our study shows that despite the abnormal T cell profile after allogeneic HSCT for SAA using the FCC regimen, this regimen is conducive to an excellent clinical outcome.

## Highlights

• Clinical outcomes are excellent using an alemtuzumab-containing hematopoietic stem cell transplantation regimen for aplastic anemia.
• Outcomes are excellent despite prolonged abnormality of the T cell profile.
• Recipient-derived CD8 T cells shape persistent mixed chimerism.

## Introduction

In contrast to hematologic malignancies, patients with nonmalignant diseases derive no benefit from graft-versus-host disease after allogeneic hematopoietic stem cell transplantation. In vivo T cell depletion (TCD) is used to reduce GVHD. We pioneered the use of alemtuzumab instead of antithymocyte globulin (ATG) for allogeneic HSCT with TCD to treat severe aplastic anemia (SAA). Using the FCC conditioning regimen of fludarabine, low-dose cyclophosphamide, and alemtuzumab (Campath-1H), we previously reported in a retrospective multicenter study an overall survival (OS) of 85% to 90% and a remarkably low incidence of chronic GVHD 1, 2. Subsequent studies have confirmed the benefit of alemtuzumab over ATG in reducing both acute and chronic GVHD in HSCT for SAA 3, 4, 5. A recent large multicenter study examining cyclophosphamide dosage deescalation in unrelated donor HSCT for SAA using ATG confirmed excellent OS but reported the 1-year incidence of chronic GVHD of 22.5% to 31.7%, depending on the cyclophosphamide dose used [6]. Allogeneic HSCT with alemtuzumab for TCD also has been applied to the treatment of sickle cell anemia, with excellent clinical outcomes reported [7].

Previous studies of chimerism post-HSCT for SAA examining unfractionated blood mononuclear cells have shown a significant incidence of mixed chimerism. Although graft failure occurred in a high proportion of patients with progressive mixed chimerism, stable mixed chimerism was associated with a low risk of chronic GVHD [8]. We have previously reported that stable mixed T cell chimerism along with full donor myeloid engraftment was frequent and persisted after cessation of post-HSCT immunosuppressive therapy [1]. This effect also has been reported in a proportion of children undergoing HSCT for SAA using the FCC regimen [9]. The very low incidence of GVHD and sustained mixed T cell chimerism suggests that a state of mutual immunologic tolerance exists after FCC HSCT.

In the present study, we investigated the recovery of lymphocyte subsets in patients with SAA who underwent HSCT at King's College Hospital using the FCC-conditioning regimen. We report the lymphocyte profile associated with excellent clinical outcomes, and the basis for the persistent coexistence of donor and recipient T cells.

## Patients and Methods

### Patients

Between 2007 and 2015, 45 consecutive patients with acquired idiopathic SAA underwent HSCT at King's College Hospital. The diagnosis of aplastic anemia (AA) [10] and disease severity 11, 12 were defined using standard criteria. Fanconi's anemia was excluded by diepoxybutane stimulation of cultured peripheral blood chromosomes. Patients with likely constitutional AA, based on an identified mutation, family history, and/or somatic anomalies but no identified mutation, were excluded. Patient characteristics are summarized in Table 1. National Research Ethics Committee approval was obtained to collect patient samples for research studies via the King's College London Haemato-Oncology Tissue Bank, and informed written consent was obtained in accordance with the Declaration of Helsinki.

### Transplantation conditioning regimen

High-resolution DNA typing for HLA-A, -B, -C, -DRB1, and -DQB1 was performed for all patients. The FCC conditioning regimen was used for 10/10 HLA-matched HSCT recipients, as described previously [1]. FCC conditioning with 2 Gy of total body irradiation was used for 9/10 HLA-matched HSCT recipients. The median alemtuzumab dose was 70 mg (range, 45 to 100 mg). Cyclosporine (CSA) alone was used as post-HSCT immunosuppressive therapy. Four patients later required the addition of mycophenolate mofetil owing to renal impairment that precluded full-dose CSA. Target trough CSA blood levels were 250 to 350 µg/L. IST was continued for 9 months post-HSCT, followed by tapering over the next 3 months, provided that hematologic parameters and mixed T cell chimerism were stable.

### Lymphocyte phenotyping

Twenty-nine patients underwent longitudinal multiparameter flow cytometry analysis of lymphocyte subset reconstitution. Patients who received prednisolone or rituximab were excluded after the initiation of treatment. Analysis of 11 healthy adult volunteers was performed as well. Cryopreserved peripheral blood mononuclear cells were thawed and labeled with fluorochrome-conjugated antibodies to characterize lymphocyte subsets. Analysis was performed by flow cytometry using a FACSCanto II or LSRFortessa flow cytometer (BD Biosciences, San Diego, CA). Results were analyzed with FlowJo software (FlowJo, Ashton, OR). Dead cells were excluded from the analysis based on their forward-scatter and side-scatter properties, and staining with the Zombie Fixable viability dye (BioLegend, San Diego, CA). Monocytes were excluded based on expression of CD14. NK cells were defined as CD3^−^/CD19^−^/CD56^+^. B cells were defined as CD19^+^. CD4 T cells were defined as CD3^+^/CD4^+^, and CD8 T cells were defined as CD3^+^/CD8^+^ with naïve (CD45RA^+^/CD27^+^/CD62L^+^), memory (CD45RA^−^/CD27^+^), effector (CD45RA^−^/CD27^−^/CD62L^−^), and terminal effector (CD45RA^+^/CD27^−^/CD62L^−^) subsets.

### Chimerism

Chimerism was assessed in unfractionated peripheral blood, CD3^+^ T cells, and CD15^+^ granulocytes. Full donor chimerism was defined as >95% donor hematopoietic cells. Lymphocyte subsets were isolated using a FACSAria II cell sorter (BD Biosciences) (purity >95%). Genomic DNA was extracted using a QIAamp DNA Micro Kit (Qiagen, Venlo, The Netherlands). The percentage of donor chimerism was determined using the PowerPlex 16 HS multiplex short tandem repeat system for DNA typing (Promega, Madison, WI). Genetic analysis was performed using an Applied Biosystems 3130xl Genetic Analyzer (Thermo Fisher Scientific, Waltham, MA), and results were interpreted using ChimeRMarker version 3.0.9 (Carolina Biosystems, Orech, Czech Republic).

### Statistical analysis

Univariate comparisons and multivariate analysis were performed using the Cox proportional hazards regression model with SPPS software (IBM, Armonk, NY). Comparisons of lymphocyte subset composition in patients and healthy volunteers were made with the Mann-Whitney *U* test using Prism version 6.0 (GraphPad Software, La Jolla, CA). All tests were 2-tailed, with a *P* value <.05 considered statistically significant. All data were censored as of December 30, 2015.

## Results

### Clinical outcomes after FCC HSCT

**Figure 1:**
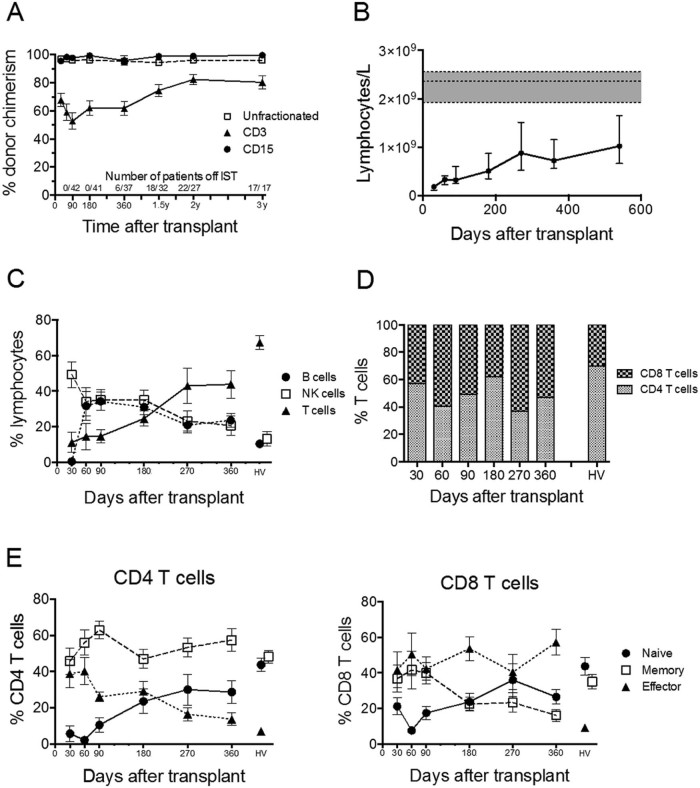
Serial analysis of peripheral blood chimerism and lymphocyte composition after FCC HSCT. (A) Percentage donor chimerism of unfractionated, purified CD3 and purified CD15 peripheral blood cells. Mean and SEM are shown. (B) Reconstitution of peripheral blood lymphocytes after FCC HSCT. Median and interquartile range are shown, and the horizontal dotted lines enclosing gray boxes represent the median and interquartile range of 11 adult healthy volunteers. Comparisons between cell numbers at each time point and healthy volunteers were performed using a 2-tailed Mann-Whitney *U* test. Lymphocyte numbers at all time points were significantly below numbers for healthy volunteers (*P* < .0005). (C) Percentage of NK, B, and T cells. Mean and SEM are shown, and values for 11 adult healthy volunteers (HV) are indicated. (D) Percentage of CD4 and CD8 T cells within the CD3 T cell population using mean values. (E) Percentages of naïve, memory, and effector subset composition of CD4 T cells (left) and CD8 T cells (right). Mean and SEM are shown, and values for 11 adult healthy volunteers (HV) are indicated.

In this single-center study, we have confirmed the excellent clinical outcomes reported previously after HSCT using FCC conditioning for SAA [1] (Table 2). The median duration of follow-up after HSCT was 31.4 months (range, 3 to 93 months), with excellent OS and event-free survival (EFS) at 5 years of 93% and 91%, respectively. Of note, there was no significant difference in outcomes for recipients of matched sibling donor transplants compared with recipients of unrelated donor transplants or for patients aged >50 years (n = 14) compared with younger patients. Three patients died, resulting in a TRM of 7% at 1 year. The rate of GVHD was very low, and the majority of cases were mild. Reliable and sustained engraftment was observed, with only 1 graft failure noted, in a patient who received a suboptimal bone marrow infusion cell dose. Sequential chimerism data were available for 42 patients (93%), which confirmed the persistent mixed T cell chimerism reported previously with the FCC conditioning regimen [1] (Figure 1A).

Epstein-Barr virus (EBV) viremia was detected in 20 patients (47%), but treatment was required in only 2 patients (5%). One patient developed biopsy-proven EBV post-transplantation lymphoproliferative disease (PTLD) on day 120, and the second patient had an excessive EBV viral load on day 66 but no signs of PTLD. Cytomegalovirus (CMV) viremia was seen in 11 patients (42%), but no patient progressed to CMV disease, owing to preemptive therapy. Adenovirus viremia was seen in 3 patients (7%). Invasive fungal infections were diagnosed in 3 patients (7%), including 2 patients with a fatal outcome, both of whom had the infection before transplantation.

After FCC HSCT, 9 patients (20%) developed autoimmune-like pathologies, including 4 patients with hemolytic anemia, 4 patients with pure red cell aplasia (1 of whom also had immune thrombocytopenia and 1 who had thyroiditis), and 1 patient with probable immune-mediated neutropenia that responded to granulocyte colony-stimulating factor (Supplementary Table S1). All 4 patients with pathology classified as warm-type autoimmune hemolytic anemia responded to prednisolone, but 3 of these patients relapsed. Two of these 3 patients responded to rituximab therapy; the other patient, who received a 9/10 HLA-matched HSCT, had refractory and fulminant hemolysis in association with severe GVHD and multiple thromboses. Among the 4 patients with pure red cell aplasia (PRCA), 3 received a major ABO mismatched transplant that was the likely cause of the PRCA. Three of these 4 patients recovered, attaining transfusion independence, whereas in 1 patient the PRCA was refractory to steroids, i.v. immunoglobulin, rituximab, and donor lymphocyte infusion. This patient underwent a second HSCT from an alternative ABO-matched unrelated donor.

### Lymphocyte reconstitution after FCC HSCT

Serial analysis of peripheral blood lymphocytes of 29 FCC HSCT recipients showed the expected lymphopenia associated with the use of alemtuzumab, with the total number of lymphocytes remaining significantly below normal (*P* < .0001) at 1 year and beyond (Figure 1B). Natural killer (NK) cells were the predominant lymphocyte population present early post-HSCT, representing 49.3% of lymphocytes at day +30 (Figure 1C). B cells were not detected at day 30 but rapidly recovered, representing 31.7% of lymphocytes at day 60 (Figure 1C), and remained significantly above normal at day 360 (23.7%, compared with 10.5% for healthy volunteers; *P* = .002%).

### T cell deficiency is prolonged, and the CD8 T cell population is skewed to an effector phenotype

T cells were profoundly deficient, representing only 11.3% of lymphocytes at day 30, and increasing to 43.8% at 1 year, but still significantly below normal (67.2%; *P* = .018) (Figure 1C). Rapid recovery of B cells with prolonged T cell deficiency produced abnormal dominance of B cells over T cells, which was maintained in the long term. The percentage of CD4 T cells in the T cell population was lower than normal, producing inversion of the usual CD4:CD8 T cell ratio (Figure 1D). Memory and effector T cells predominated early post-HSCT (Figure 1E). Naïve T cells began to recover after 6 months (Figure 1E). The proportions of naïve, memory, and effector subsets within the CD4 T cell population were near normal at 1 year (Figure 1E, left). In contrast, CD8 T cell subset composition remained abnormal at 1 year owing to a high proportion of effector T cells (57.2%, compared with 9.3% for healthy volunteers; *P* < .0001) (Figure 1E, right).

### Persistence of recipient CD8 T cells after FCC HSCT

**Figure 2:**
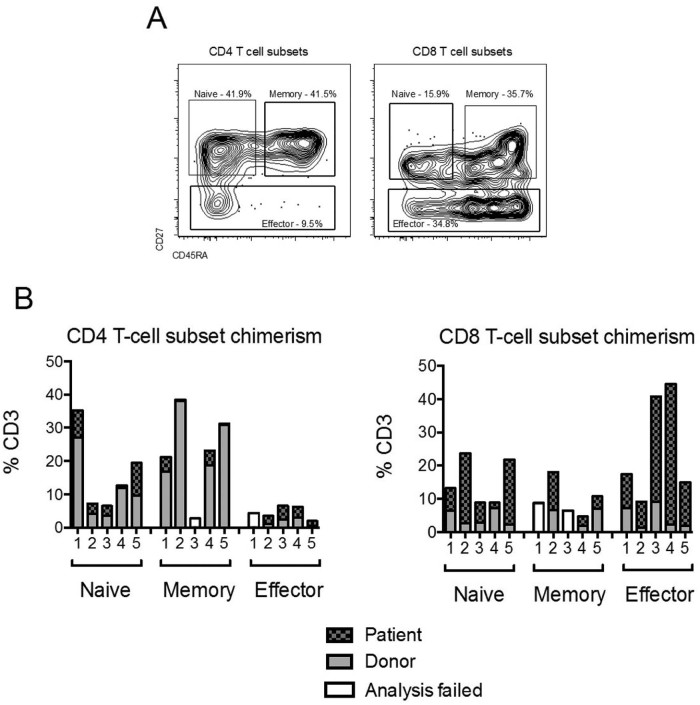
Analysis of individual patients showed sustained mixed T cell chimerism at 1 year after FCC HSCT is due primarily to persistence of recipient CD8 T cells, with notable contribution of the effector subset. (A) Panels illustrate the gating strategy used for isolation of naïve, memory, and effector T cell subsets by FACS. (B) CD4 T cell (left) and CD8 T cell (right) subset chimerism at 1 year for 5 patients. CMV serostatus of donor/recipient and reactivation early post HSCT: patient 1, negative/negative; patient 2, negative/positive no reactivation; patient 3, positive/negative and reactivation; patient 4, negative/positive and reactivation; patient 5, negative/positive no reactivation.

Lymphocyte subsets from 5 patients with mixed T cell chimerism at 1 year were isolated by fluorescence-activated cell sorting, and donor/recipient composition was determined by analysis of informative alleles from polymorphic short tandem repeat loci. Four patients had >93% donor NK cells and >76% donor B cells. Patient 3 had only 39% donor NK cells and 76% donor B cells. Naïve, memory, and effector T cell subsets were isolated using the gating strategy shown Figure 2A. The results shown in Figure 2B show that the mixed T cell chimerism at 1 year was due principally to the persistence of recipient CD8 T cells. The median percentage contribution to recipient CD3 chimerism was 37.9% for the CD8 T cell population and 8.5% for the CD4 T cell population (*P* = .008). Recipient chimerism was detected in all CD8 T cell subsets, but with a notable contribution of the effector subset in patients 3 and 4, both of whom had CMV reactivation early after HSCT. A significant correlation was observed between lower donor T cell chimerism at 1 year and CMV reactivation or EBV viremia early after HSCT. Patients who were donor and/or recipient CMV/EBV seropositive but had no viremia post-HSCT had a median of 77% donor CD3 chimerism, compared with 57% donor CD3 chimerism in patients who had viremia post-HSCT (*P* = .036). Follow-up analysis of patients 1, 2, and 3 at >2 years post-HSCT showed that their T cell subset mixed chimerism profile remained stable after IST cessation, and that the numbers of CD4 and CD8 T cells did not increase (data not shown).

## Discussion

Using the FCC conditioning regimen for allogeneic HSCT to treat SAA at a single center and with long-term follow up, we have confirmed the excellent clinical outcomes reported in our previous multicenter study [5]. The incidence of chronic GVHD was remarkably low, particularly given the high proportion of matched unrelated donors in this cohort. The comparable outcome of older (age >50 years) and younger patients is also encouraging, given that increasing age is typically associated with worse outcomes [13]. Rapid full donor myeloid engraftment occurred, and there was only 1 case of graft failure, associated with a low infused bone marrow stem cell dose. The majority of patients exhibited sustained mixed T cell chimerism that persisted despite withdrawal of immunosuppression at 1 year after transplantation.

The alemtuzumab-containing FCC regimen caused profound and prolonged T cell depletion, particularly in CD4 T cells, which recovered more slowly than CD8 T cells. The small numbers of T cells that survived depletion with alemtuzumab were almost exclusively of the memory or effector subsets and dominated the circulating population for at least 6 months. We previously reported the same selective preservation of antigen-experienced T cells after HSCT using a fludarabine, busulfan, and alemtuzumab regimen for treatment of patients with hematologic malignancies [14]. The very slow recovery of T cells is likely due to thymic atrophy in adults, which limited de novo production of naïve T cells after HSCT. The combination of Tcell depletion and the relatively mild conditioning with fludarabine and low-dose cyclophosphamide, which is unlikely to induce a strong cytokine storm, limits the potential for T cell alloreactivity. Of the 7 patients who developed GVHD, 6 had >90% donor CD3 chimerism and 1 had 75% donor CD3 chimerism by day 60.

Mixed T cell chimerism is frequent after reduced-intensity conditioning and particularly common after TCD with alemtuzumab [15]. Our investigation of mixed chimerism within T cell subsets found that CD4 T cells were mainly donor-derived by 1 year but that CD8 T cells remained predominantly recipient-derived. Although naïve, memory, and effector subset frequencies in the CD4 T cell population were near normal by 1 year, numbers were low in all patients, and deficiencies persisted after cessation of IST. In contrast, recovery of CD8 T cells was much more robust, but an atypical composition developed owing to expansion of the effector CD8 T cell subset, which rose from a mean of 17.6% CD8 T cells at day +90 to 57.2% at 1 year (compared with 9.3% for healthy volunteers). Recipient cells were present in all CD8 T cell subsets but was notably high in the effector population, particularly for the 2 patients who experienced CMV reactivation early after HSCT.

Expansion of the effector CD8 T cell population occurred despite continued IST for at least 1 year. CMV reactivation and EBV viremia early after transplantation were correlated with higher recipient CD3 chimerism at 1 year; thus, we speculate that the expansion of effector CD8 T cell numbers may be due to antigen-driven proliferation of pathogen-specific recipient CD8 T cells. Limited sample availability and low T cell numbers precluded our investigation of the antigen specificity of the effector CD8 T cells. The incidence of viral disease post-FCC was low, despite EBV and CMV viremia in 47% and 42% of patients, respectively. Recipient-derived effector CD8 T cells may confer a degree of antivirus immunity after FCC HSCT, despite continued IST. Other recent studies have reported that virus-specific CD8 T cells can have a substantial impact on T cell composition after HSCT. Massive expansion of CMV-specific effector CD8 T cells is seen following CMV reactivation after HSCT and has a long-term impact on T cell reconstitution 16, 17. In the setting of TCD using alemtuzumab-based reduced-intensity conditioning in patients undergoing transplantation for hematologic malignancies, Sellar et al. showed that CMV-specific CD8 T cells were of recipient origin and contributed significantly to higher recipient chimerism [18].

For patients with hematologic malignancies, routine clinical practice is to administer donor lymphocyte infusion (DLI) for mixed T cell chimerism to promote conversion to full donor chimerism and prevent disease relapse [19]. DLI is not administered to patients with SAA after FCC HSCT because disease relapse does not occur and DLI has the associated risk of GVHD. Our study of lymphocyte composition and chimerism provides novel insight into the long-term impact of HSCT conditioning with alemtuzumab. Stable coexistence of donor and recipient T cells for many years after withdrawal of immunosuppression, reliable engraftment, and low rates of GVHD indicates that a state of mutual tolerance exists after allogeneic FCC HSCT. HLA-matching and T cell depletion using alemtuzumab together with IST for at least 1 year after HSCT is an effective strategy to minimize alloreactivity. Although our study shows that the FCC HSCT regimen results in an atypical lymphocyte composition, clinical course and outcomes for patients are remarkably good.

The only complication consistently observed after FCC HSCT is emergence of autoimmune-like diseases that are predominantly antibody-mediated cytopenias, seen in 6 patients (14%) in this study. Secondary autoimmune disorders commonly occur after treatment with alemtuzumab in other clinical settings [20]. Prolonged T cell deficiency may contribute to dysregulated B cell behavior. We previously reported that clonal gammopathies are frequent after alemtuzumab-based HSCT [21]. No association of autoimmune-like disease with circulating B cells or other lymphocyte populations was found in our study (data not shown).

In conclusion, despite persistent T cell deficiency, excellent survival and low incidence of viral disease was observed after FCC HSCT for SAA. The very low incidence of GVHD with long-term stable mixed T cell chimerism, shaped by recipient CD8 T cells, in the absence of immunosuppression indicates that FCC conditioning favors mutual tolerance.

## Figures and Tables

**Table 1:** Patient Pretransplantation Characteristics

Characteristic	Value
Number of patients	45
Age, yr, median (range)	32 (15-63)
Age >50 yr, n (%)	14 (31.1)
Males/females, n	28/17
Etiology of aplasia, n (%)	
Idiopathic	41 (91.2)
Posthepatitic (seronegative)	2 (4.4)
Eosinophilic fasciitis	1 (2.2)
Celiac disease	1 (2.2)
Type of donor, n (%)	
Matched sibling	12 (26.6)
Unrelated	33 (73.4)
9/10 unrelated	8 of 33 (24.2)
Previous immunosuppressive therapy, n/N (%)	
Matched sibling donor	6/12 (50)
Unrelated donor	27/33 (81.8); *P* = .055
Time to transplantation, d, median (range)	
Matched sibling donor	5.9 (2.7-34.7)
Unrelated donor	8.4 (2.1-178.9); *P* = .095
HLA alloimmunization, n (%)	11 (24.4)
Stem cell source, n (%)	
Bone marrow	7 (15.5)
Peripheral blood stem cells	38 (84.5)
PNH clone, n (%)	21 (46.6)
PNH clone size, % median (range)	
Granulocytes	2 (0.02-40)
Monocytes	2.85 (0.01-32)
Red cells	Not available
Alemtuzumab dose, mg, median (range)	70 (45-100)
CD34^+^ stem cell dose, × 10^6^/kg, median (range)	6.55 (1.97-12.40)
Follow-up after HSCT, mo, median (range)	31.4 (3-93)

HSCT indicates hematopoietic stem cell transplantation; PNH, paroxysmal nocturnal hemoglobinuria.

**Table 2:** Patient Outcomes After FCC HSCT

Outcome	Value
Time to neutrophil count >0.5 × 10^9^/L, d, median (range)	12 (10-22)
Time to platelet count >20 × 10^9^/L, d, median (range)	12 (9-61)
Primary graft failure, n (%)	1 (2.2)
Acute GVHD, n (%)	6 (13.3)
Grade I/II	6
Grade III/IV	0
Chronic GVHD, n (%)	6 (13.3)
Mild	4
Moderate	1
Severe	1
1-year TRM, n (%)	3 (6.6)
5-year OS, %	93.1
5-year EFS, %	90.7
5-year EFS, MSD (n = 12) versus UD (n = 33), %	100 versus 87.4 (*P* = .219)
5-year EFS, age ≤50 yr (n = 31) versus >50 yr (n = 14), %	93 versus 85.7 (*P* = .356)

FCC indicates fludarabine, cyclophosphamide, and alemtuzumab (Campath-1H); HSCT, hematopoietic stem cell transplantation; GVHD, graft-versus-host disease; TRM, transplantation-related mortality; OS, overall survival; EFS, event-free survival; MSD, matched sibing donor; UD, unrelated donor.

## Supplementary Data

Table S1 Autoimmune-like disorders and other events after FCC HSCT. Table S1
