# Supplementary material for: Mixed T Cell Chimerism After Allogeneic Hematopoietic Stem Cell Transplantation for Severe Aplastic Anemia Using an Alemtuzumab-Containing Regimen Is Shaped by Persistence of Recipient CD8 T Cells
Source: Biol Blood Marrow Transplant. 2017 Feb;23(2):293–9. doi: 10.1016/j.bbmt.2016.11.003 (PMC5270460; doi:10.1016/j.bbmt.2016.11.003)
Supplement: Table S1 — Autoimmune-like disorders and other events after FCC HSCT. [file mmc1.docx]

**Supplementary Table 1: Autoimmune-like disorders and other events after FCC HSCT**

|  | **N** | **Date diagnosis**  **post-transplant** | **Post-transplant features** | **Treatment and outcome** | **Latest chimerism** |
| --- | --- | --- | --- | --- | --- |
| Autoimmune –like haemolytic anaemia | 4 | 1.D+270  2.D+180  3.D+210  4. D+180 | 1.DAT IgG4+,C3D3+  2.DAT IgG3+, C3D 4+  3.IgG4+, C3D4+  4. IgG3+, C3D4+ | 1.Prednisolone: CR, relapse, Rituximab: CR  2.Prednisolone: CR, relapse, NR to Rituximab, splenectomy, plasma exchange  3.Prednisolone: CR, relapse, Rituximab:CR  4. Prednsiolone: CR | 1.CD15 98%, CD3 66%  2.CD15 98%, CD3 98%  3.CD15 100%, CD3 92% |
| Pure red cell aplasia (PRCA) | 2 | N/A* | Major ABO m/m; rHuEpo, IVIg  Major ABO m/m; IVIg | CR, day + 164  CR, day +1240 | CD15 99%, CD3 51%  CD15 86%, CD3 83% |
| PRCA + thyroiditis | 1 | AIT:D+360 | Major ABO m/m; | IVIg, Rituximab x 2, Epo, DLI to 10^7^ CD3/kg NR, day + 1147 | CD15 99%, CD3 91% |
| PRCA+ITP | 1 | ITP:D+224 | Minor ABO m/m; | Epo, IVIg, continued CSA:PR | CD15 100%, CD3 24% |
| ITP | 1 | D+670 | Moderate thrombocytopenia | No treatment required |  |
| Probable immune-mediated neutropenia | 1 | D+291 | Anti-neutrophil Abs negative, BM normal granulopoiesis, no other cause for neutropenia | GCSF: CR, day+ | CD15 100%, CD3 100% |
| Good’s syndrome pre-HSCT  (Thymoma and CVID: Pre-HSCT, IgG 4.1 (NR 6.34-18.11), IgA 0.3 (NR 0.87-4.12), IgM 0.1 g/l (NR 0.53-2.23). | 1 | Pre-HSCT | 6 months post HSCT, IgG 5.5, IgA 0.9, IgM 0.9g/L, CD4 64 cells/ml (NR 404-1612), CD8 29 (NR 220-1129) with CD15 100%, CD19 100% and CD3 26%. At 13 months post HSCT, IgG 4.3, IgA 0.28, IgM 0.3g/l, CD4 189, CD8 41 cells/ml. | Transient B cell recovery | CD15 99%, CD3 78%  IgG 4.54, IgA 0.09, IgM 0.11g/l |
| Dapsone-induced pancytopenia | 1 | D+344 | BM trilineage dysplasia, PB chimerism: CD15 95% and CD15 53% chimerism | Normal FBC and BM morphology on withdrawal of dapsone | CD15 99%, CD3 53% |
| Probable MM- induced pancytopenia | 1 | D+150 | BM hypocellular (40% cellularity), foamy macrophages and stromal degeneration, PB chimerism: CD15 100%, CD3 96% | Recovery of FBC on withdrawal of MMF | CD15 100%, CD3 84% |
| Pregnancy | 1  1 | Conceived  on D+1095 | Female  Male | Delivered healthy baby day + 1095  Fathered healthy baby, conceived approx. 2 years post HSCT | CD15 86%, CD3 83% |
